# Nonlinear duration–efficacy association of neoadjuvant PD-1/PD-L1 blockade with chemoradiotherapy in pMMR/MSS locally advanced rectal cancer: a systematic review and meta-analysis

**DOI:** 10.3389/fonc.2026.1855615

**Published:** 2026-09-09

**Authors:** Shuyang Hu, Xia Zhang, Chenyang Ge, Shicheng Zhou, Jing Ba, Jianping Wang, Jinlin Du, Cheng Cai

**Affiliations:** 1 Department of Colorectal and Anal Surgery, Affiliated Jinhua Hospital, Zhejiang University School of Medicine, Jinhua, China; 2 Department of Oncology, Affiliated Jinhua Hospital, Zhejiang University School of Medicine, Jinhua, China

**Keywords:** complete response, immunotherapy, locally advanced rectal cancer, neoadjuvant chemoradiotherapy, PD-1 blockade, PD-L1 blockade

## Abstract

**Background:**

Neoadjuvant PD-1/PD-L1 combined with chemoradiotherapy (CRT) has shown encouraging complete response (CR) rates in proficient mismatch repair (pMMR) or microsatellite-stable (MSS) locally advanced rectal cancer (LARC), yet immunotherapy duration varies widely across trials and its interaction with radiotherapy fractionation remains unclear.

**Methods:**

PubMed, Embase, and Cochrane Library were searched through October 8, 2025 for prospective trials of PD-1/PD-L1 inhibitors plus CRT in pMMR/MSS LARC. Random-effects models pooled CR (cCR+pCR) rates, with subgroup analyses and study-level meta-regression conducted to explore sources of heterogeneity. A linear probit model and an interactive quadratic probit model were applied to assess associations between treatment duration and response (PROSPERO number CRD420251253156).

**Results:**

Eighteen trials (972 patients; 10 LCRT-based and 8 SCRT-based) were included. Pooled CR and pCR rates were 44% (95% *CI* 0.38–0.49) and 40% (95% *CI* 0.36–0.45). CR was higher with SCRT plus ICI than LCRT plus ICI (50% vs 39%; *P* = 0.03), and pCR similarly favored SCRT (47% vs 34%; *P* < 0.01). Meta-regression identified radiotherapy fractionation and immunotherapy duration as study-level factors associated with CR. In analyses restricted to randomized controlled trials (RCTs), longer immunotherapy duration was positively associated with response. Exploratory quadratic models using all eligible studies suggested an inverted U-shaped pattern for LCRT (modeled peak at approximately 4.4 months) and a U-shaped pattern for SCRT (modeled trough at approximately 2.5 months).

**Conclusion:**

Among the included RCTs, longer immunotherapy duration was associated with higher response rates. The nonlinear patterns observed in the combined dataset were exploratory, may reflect heterogeneity between studies, and require prospective validation before informing treatment duration.

**Systematic review registration:**

https://www.crd.york.ac.uk/PROSPERO/view/CRD420261348026, identifier CRD420251253156.

## 1 Introduction

Neoadjuvant immunotherapy has transformed the management of deficient mismatch repair (dMMR) or microsatellite instability–high (MSI-H) rectal cancer, producing unprecedented complete response (CR) rates in multiple prospective studies (1–5). However, this subtype accounts for less than 10% of rectal cancers (6). The vast majority of patients harbor proficient mismatch repair or microsatellite-stable (pMMR/MSS) tumors, a population that derives limited benefit from immune checkpoint inhibitors (ICIs) alone because of their intrinsically immune-quiescent tumor microenvironment (7–9). Radiotherapy (RT) is known to enhance antigen release, dendritic-cell activation, and T-cell priming, thereby providing a compelling biological rationale for combining ICIs with chemoradiotherapy (CRT) in pMMR/MSS locally advanced rectal cancer (LARC) (10, 11).

Randomized trials in resectable lung cancer and early triple-negative breast cancer have shown that neoadjuvant or perioperative regimens containing ICIs can improve pathological response or event-free outcomes (12–14). These findings provide broader support for investigating ICIs in the neoadjuvant setting but cannot be directly extrapolated to pMMR/MSS rectal cancer. Separately, an international multicenter registry study in rectal cancer reported long-term outcomes of watch-and-wait management among patients who achieved a clinical complete response after neoadjuvant treatment, although it was not specific to immunotherapy (15). Prospective trials in pMMR/MSS LARC have reported encouraging but variable CR rates after CRT combined with ICIs (16–33). Nevertheless, the available evidence remains preliminary and heterogeneous, and mature survival outcomes have not yet been reported. Treatment strategies also vary across trials. ICIs have been combined with either long-course RT (LCRT) or short-course RT (SCRT), and immunotherapy duration ranges from 6 to 24 weeks (16–33). Emerging evidence from dMMR/MSI-H disease indicates that longer pretreatment immunotherapy exposure may be associated with higher CR rates (34), but whether this association extends to pMMR/MSS tumors remains uncertain. Previous meta-analyses have demonstrated improved CR or tumor regression with CRT-ICI combinations, and some suggest differences in efficacy between SCRT- and LCRT-based regimens (35, 36). However, these analyses have uniformly treated immunotherapy duration as a categorical variable, precluding the evaluation of continuous, nonlinear duration–response associations. Understanding how treatment duration interacts with radiation fractionation is particularly important because SCRT and LCRT represent fundamentally distinct radiobiological environments. LCRT delivers protracted low-dose fractions that may induce chronic antigen exposure, lymphocyte depletion, and T-cell exhaustion (37, 38), whereas SCRT provides an intense burst of immunogenic cell death and rapid dendritic-cell activation (39–41). These intrinsic differences imply that the optimal window for immunotherapy efficacy may not only be nonlinear but may also differ between RT regimens. Yet, no study has systematically quantified these interactions across available clinical trials. We therefore conducted a systematic review and meta-analysis to examine this association in pMMR/MSS LARC and explore its potential interaction with radiotherapy fractionation. The nonlinear analyses were intended to generate hypotheses for prospective validation rather than define optimal treatment durations.

## 2 Methods

This systematic review and meta-analysis was conducted in accordance with the Preferred Reporting Items for Systematic Reviews and Meta-Analyses (PRISMA) guidelines. The study protocol was prospectively registered in PROSPERO (CRD420251253156).

### 2.1 Search strategy and selection criteria

We systematically searched PubMed, Embase and Cochrane Library from database inception to October 8, 2025. Search terms included Medical Subject Heading (MeSH) and free-text keywords related to rectal cancer, neoadjuvant therapy, and immune checkpoint inhibitors. Boolean operators (“AND” and “OR”) were used to combine search components, including but not limited to: (Rectal Neoplasms) AND (Neoadjuvant Therapy) AND (Immunotherapy OR Immune Checkpoint Inhibitors). No language or publication restrictions were applied. The complete search strategies for each database are provided in the **Supplementary Materials**.

Articles retrieved from PubMed, EMBASE, and Cochrane Library were exported to EndNote, where duplicates were identified and removed. Two investigators (SH and XZ) independently screened studies by title and abstract, followed by a sequential full-text review of potentially eligible trials to confirm relevance. Any disagreements or discrepancies arising during either screening process were resolved through consultation with a third investigator (CG).

Studies were eligible if they met all of the following inclusion criteria: (1) prospective phase II-III clinical trials; (2) enrollment of patients with pMMR/MSS LARC; (3) use of neoadjuvant CRT in combination with PD-1 or PD-L1 inhibitors, followed by surgery or watch-and-wait strategy; (4) reporting of pCR and/or overall CR rates. Exclusion criteria were: (1) non-original studies including retrospective or observational studies, reviews or preclinical investigations; (2) use of concomitant investigational agents other than ICIs, such as targeted therapies, cancer vaccines, or immunomodulators; (3) absence of MMR/MSI testing; (4) studies exclusively enrolling patients with dMMR/MSI-H tumors; (5) failure to report the number of pMMR/MSS patients or to provide response outcomes for this subgroup.

Two investigators (SH and XZ) independently screened full-text articles and extracted all relevant data. Discrepancies were resolved by consensus or through consultation with a third reviewer (CG). Extracted data included: (1) study characteristics: trial identifier, first author, year of publication or presentation and study phase; (2) patient and outcome data: cohort proportions of baseline tumor burden indicators (including cT4, cN2, cN+, EMVI+ and MRF/CRM+), number of pMMR/MSS patients included in the CR analysis, and numbers achieving CR or pCR; (3) treatment details: ICI class and regimen, radiotherapy modality (LCRT or SCRT), chemotherapy backbone, treatment schedule, and median duration of immunotherapy (months). The duration of immunotherapy was calculated based on the planned treatment schedules indicated in each study’s protocol. For standardization, one month was defined as 28 days (4 weeks). Data were organized and recorded in a standardized Excel sheet to facilitate consistent comparison and synthesis across studies.

### 2.2 Data analysis

Publication bias was examined using funnel plots and Egger’s linear regression test. The quality of the included studies was rigorously evaluated using two validated assessment tools: the Cochrane Risk of Bias tool version 2 (RoB-2) for randomized controlled trials (42), as well as the Methodological Index for Non-randomized Studies (MINORS) for single-arm interventional studies and non-randomized controlled trials (43).

All statistical analyses were conducted using R (version 4.4.3). Pooled CR and pCR rates were estimated using a random-effects model with 95% confidence intervals (CIs). Forest plots were generated to provide a visual representation of the pooled effect sizes. Additionally, a sensitivity analysis was conducted via the sequential omission of individual studies to evaluate the robustness of the combined results. Heterogeneity was assessed using the *I*² statistic; values of 0–40% might not be important, 30–60% may represent moderate heterogeneity, 50–90% substantial heterogeneity, and 75–100% considerable heterogeneity (44). Exploratory subgroup analyses were conducted according to radiotherapy strategy, PD-1/PD-L1 inhibitor type, and treatment duration. A two-sided *P* < 0.05 was considered statistically significant. To further explore potential sources of heterogeneity, study-level univariate meta-regression analyses were performed.

To evaluate the duration-response relationship, a linear probit model restricted to randomized controlled trials (RCTs) was first applied to assess the linear association between immunotherapy duration and CR rates. To accommodate all eligible studies and explore nonlinear association, an interactive quadratic probit model was subsequently fitted, incorporating treatment duration, radiotherapy type (LCRT vs SCRT), and their interaction. Model adequacy was assessed using a goodness-of-fit test (*P*>0.05), and the significance of the interaction was evaluated with a likelihood ratio test. Predicted CR–duration curves were generated for each radiotherapy regimen to illustrate the resulting nonlinear trajectories. To assess the robustness of the findings, particularly those from the nonlinear models, a leave-one-out (LOO) sensitivity analysis was performed.

### 2.3 Role of the funding source

The funder of the study had no role in study design, data collection, data analysis, data interpretation, or writing of the report.

## 3 Results

**Figure 1:**
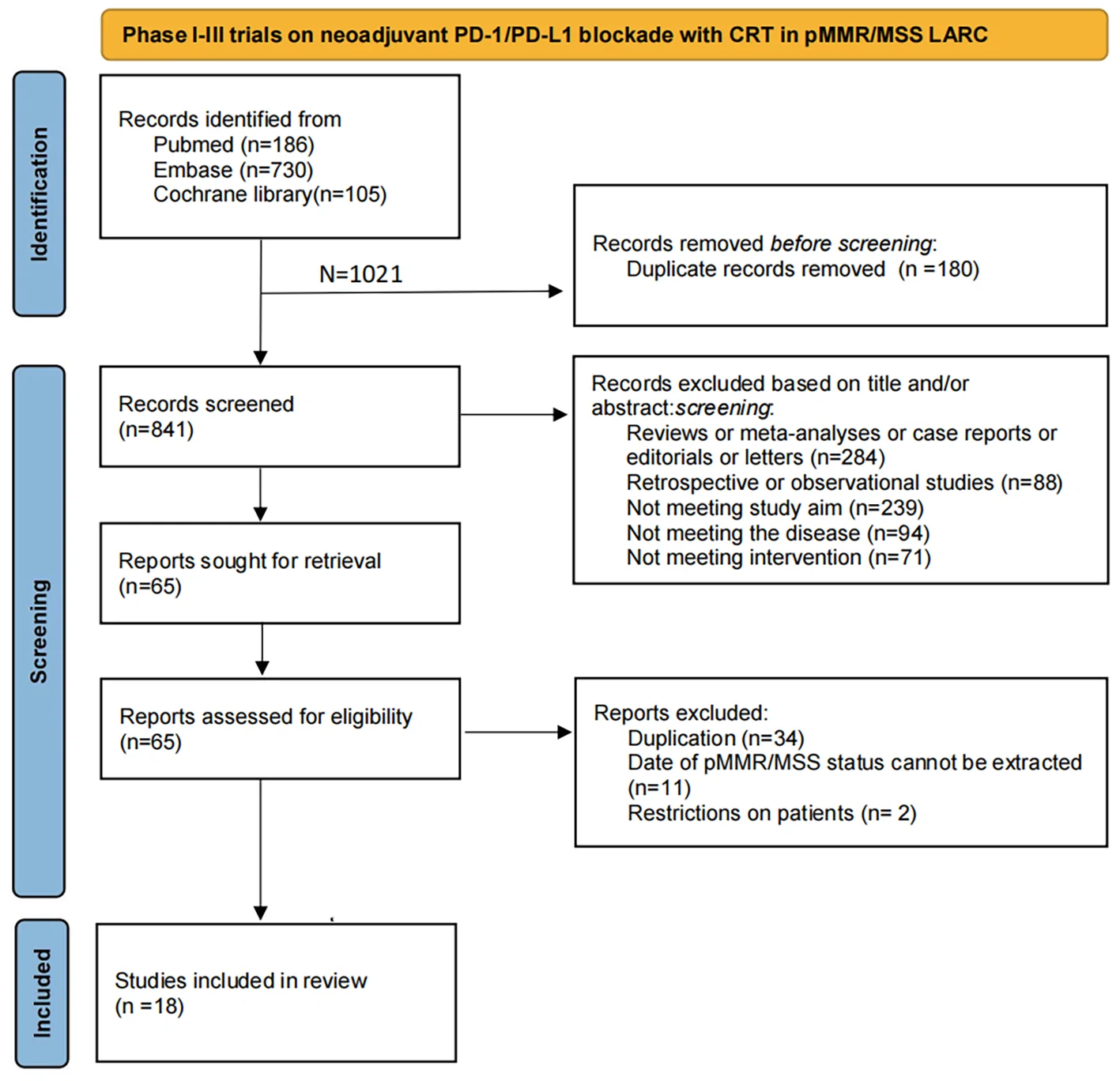
PRISMA 2020 flowchart. PRISMA indicates Preferred Reporting Items for Systematic Reviews and Meta-Analyses; CRT, chemoradiotherapy; pMMR/MSS, proficient mismatch repair/microsatellite-stable; LARC, locally advanced rectal cancer; PD-1, programmed death-1; PD-L1, programmed death-ligand 1.

The initial search across the specified databases, including PubMed, Embase, and the Cochrane Central Library, yielded 1021 records. After removing 180 duplicates, 841 records were screened by title and abstract. Of these, 776 were excluded based on the eligibility criteria, leaving 65 studies for full-text assessment. Ultimately, 18 clinical trials involving 972 patients were included in the final analysis. Two trials reported more than one eligible treatment arm, resulting in 20 treatment arms analyzed separately. The included studies comprised 6 randomized controlled trials (RCTs) (23, 25–29) and 12 single-arm phase II trials (16–22, 24, 30–33) evaluating various strategies integrating immunotherapy with radiotherapy-based regimens. Across all included studies, cCR and pCR were the primary efficacy endpoints. The funnel plots revealed no evidence of publication bias (**Figure 1**). The study selection process is summarized in the PRISMA flow diagram (**Figure 1**).

The key eligibility criteria for each clinical trial are summarized in **Table 1**. Strategies for combining ICIs with neoadjuvant CRT varied across studies. Neoadjuvant ICIs were administered in combination with LCRT in 11 treatment arms (55%) and with SCRT in 9 arms (45%). The duration of immunotherapy varies widely, ranging from 6 to 24 weeks, and PD-1 inhibitors were used in 13 treatment arms (65%). Trial phase and details of treatment administration are provided in **Table 1**. Because watch-and-wait strategies for cCR were applied inconsistently across trials (permitted in some trials but not others), the overall CR rate was selected as a more consistent primary endpoint across all included studies.

**Table 1:** Studies included in the meta-analysis to analyze the association of neoadjuvant immune checkpoint inhibitors duration with complete response rate in mismatch repair proficient locally advanced rectal cancer.

Study	cT4 (%)	cN2 (%)	cN+ (%)	EMVI+ (%)	MRF/CRM+(%)^a^	NCT	Treatment regimen	Treatment details	RT	Outcome	Median duration of treatment (months)^b^	Number of MSS patients for pCR analysis^c^	Number of pCR	Number of cCR	Number of MSS patients for CR analysis	Number of MSS patients with CR^d^	CR rate (%)
PRECAM	12.5	18.8	59.4	34.4	12.5	NCT05216653	aPD-L1	Envafolimab	SCRT	pCR	1.5	32	20	NA	32	20	62.5
UNION-III	30.1	45.1	87.6	52.2	47.8	NCT04928807	aPD-1	Camrelizumab	SCRT	pCR/cCR	1.5	105	42	0^e^	105	42	40.0
Xiao WW	52.2	34.3	85.1	55.2	43.3	NCT04304209	aPD-1	Sintilimab	LCRT	pCR/cCR	3	57	21	9^f^	67	30	44.8
NECTAR	20.0	26.0	64.0	24.0	80.0	NCT04911517	aPD-1	Tislelizumab	LCRT	pCR/cCR	2.25	46	20	1^g^	47	21	44.7
TORCH group A	16.1	43.5	93.5	22.6	27.4	NCT04518280	aPD-1	Toripalimab	SCRT	pCR/cCR	4.5	40	20	16^h^	62	36	58.1
TORCH group B	18.6	52.5	86.4	28.8	39.0	NCT04518280	aPD-1	Toripalimab	SCRT	pCR/cCR	4.5	34	17	16^i^	59	33	55.9
PKUCH 04	NA	76.0	NA	80.0	60.0	NCT04340401	aPD-1	Camrelizumab	LCRT	pCR	2.25	21	7	4^j^	25	11	44.0
PANDORA	16.7	NA	79.6	17.7	46.5	NCT04083365	aPD-L1	Durvalumab	LCRT	pCR	3	47	17	0^k^	47	17	36.2
VOLTAGE-A	12.8	NA	23.1	25.6	7.7	NCT02948348	aPD-1	Nivolumab	LCRT	pCR/cCR	2.5	38	11	0^l^	39	11	28.2
UNION-II	26.7	33.3	86.7	40.0	70.0	NCT04231552	aPD-1	Camrelizumab	SCRT	pCR	1.5	26	12	NA	26	12	46.2
Averectal	5.0	75.0	90.0	NA	NA	NCT03503630	aPD-L1	Avelumab	SCRT	pCR	3	40	15	NA	40	15	37.5
POLARSTAR experiment groups A	28.8	44.1	89.8	49.2	33.9	NCT05245474	aPD-1	Tislelizumab	LCRT	pCR	2.25	54	13	NA	54	13	24.1
POLARSTAR experiment groups B	21.8	49.1	85.5	40.0	29.1	NCT05245474	aPD-1	Tislelizumab	LCRT	pCR	2.25	48	15	NA	48	15	31.2
SPRING-01	34.7	42.9	85.7	77.6	49.0	ChiCTR2100052288	aPD-1	Sintilimab	SCRT	pCR	4.5	41	24	NA	41	24	58.5
STELLAR II	18.2	62.7	92.7	48.2	52.7	NCT05484024	aPD-1	Sintilimab	SCRT	pCR/cCR	3	72	31	19	110	50	45.5
NSABP FR-2	NA	NA	NA	NA	NA	NCT03102047	aPD-L1	Durvalumab	LCRT	pCR/cCR	2	45	10	0^m^	45	10	22.2
ESTIMATE	21.4	46.4	82.1	35.7	32.1	ChiCTR2400079718	aPD-L1	Envafolimab	LCRT	pCR/cCR	3.5	21	8	7^n^	28	15	53.6
PANFORTE	NA	NA	NA	NA	NA	NCT06099951	aPD-1	Serplulimab	LCRT	pCR/cCR	5	9	4	11	20	15	75.0
DUREC	22.0	53.0	NA	60.0	NA	NCT04293419	aPD-L1	Durvalumab	LCRT	pCR	6	56	22	NA	56	22	39.3
Jing C	NA	NA	NA	NA	NA	NCT06765616	aPD-L1	Adebrelimab	SCRT	pCR	4.5	21	10	NA	21	10	47.6

cT, clinical T; cN, clinical N; EMVI, extramural vascular invasion; MRF, mesorectal fascia; CRM, circumferential resection margin; RT, radiotherapy; m, month; aPD-1, anti-programmed death-1; aPD-L1, anti-programmed death-ligand 1; pMMR, proficient mismatch repair; MSS, microsatellite stable; pCR, pathological complete response; cCR, clinical complete response; CR, complete response; SCRT, short-course radiotherapy; LCRT, long-course radiotherapy; NA, not available.

^a^ Baseline MRF involvement and positive CRM were combined for analysis. In the context of pre-treatment assessment for rectal cancer, these terms refer to the same anatomical boundary (the mesorectal fascia) with tumor distance ≤ 1 mm.

^b^ The duration of immunotherapy was calculated based on the planned treatment schedules indicated in each study’s protocol and one month was defined as 4 weeks.

^c^ Number of MSS patients for pCR analysis was calculated as the number of patients who underwent surgery.

^d^ Number of MSS patients with CR was calculated as the number of patients with pCR, confirmed by pathological examination after surgery, and cCR, patients who achieved cCR after neoadjuvant therapy and underwent a watch-and-wait approach.

^e^ 2 patients with cCR were excluded because MSS/pMMR status was not reported.

^f^ 2 of the 11 cCR patients underwent surgery and the remaining 9 chose watch-and-wait.

^g^ 17 of 18 cCR patients underwent surgery and the remaining one chose watch-and-wait.

^h^ 11 of 27 cCR patients underwent surgery and the remaining 16 chose watch-and-wait.

^i^ 5 of 21 cCR patients underwent surgery and the remaining 16 chose watch-and-wait.

^j^ 8 of 12 cCR patients underwent surgery and the remaining 4 chose watch-and-wait.

^k^ All 6 cCR patients underwent surgery.

^l^ All 8 cCR patients underwent surgery.

^m^ All 14 cCR patients underwent surgery.

^n^ 5 of 12 cCR patients underwent surgery and the remaining 7 chose watch-and-wait.

**Figure 2:**
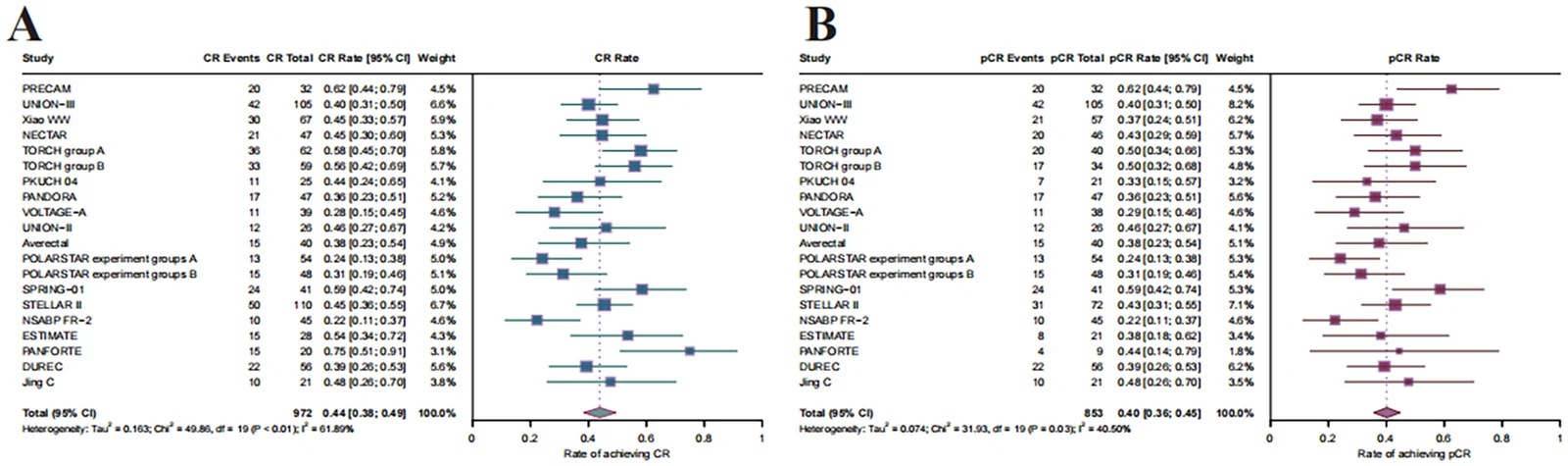
Pooled CR and pCR Rates With Neoadjuvant PD-1/PD-L1 Blockade Plus CRT in Patients With pMMR/MSS LARC. **(A)** Pooled CR rate. **(B)** Pooled pCR rate. For each study, squares represent the point estimate of the response rate, horizontal lines indicate the 95% *CI*, and the size of the square reflects the weight of the study in the meta-analysis. Diamonds represent pooled estimates with corresponding 95% CIs. Between-study heterogeneity was assessed using Tau², Cochran’s Q (Chi²) test, and the I² statistic. Subgroup differences were evaluated using Chi² tests, with corresponding *P* values reported in each panel. CR (cCR or pCR) indicates complete response (clinical complete response or pathological complete response); pCR, pathological complete response; CRT, chemoradiotherapy; pMMR/MSS, proficient mismatch repair or microsatellite-stable; LARC, locally advanced rectal cancer; PD-1, programmed death-1; PD-L1, programmed death-ligand 1; *CI*, confidence interval.

**Figure 3:**
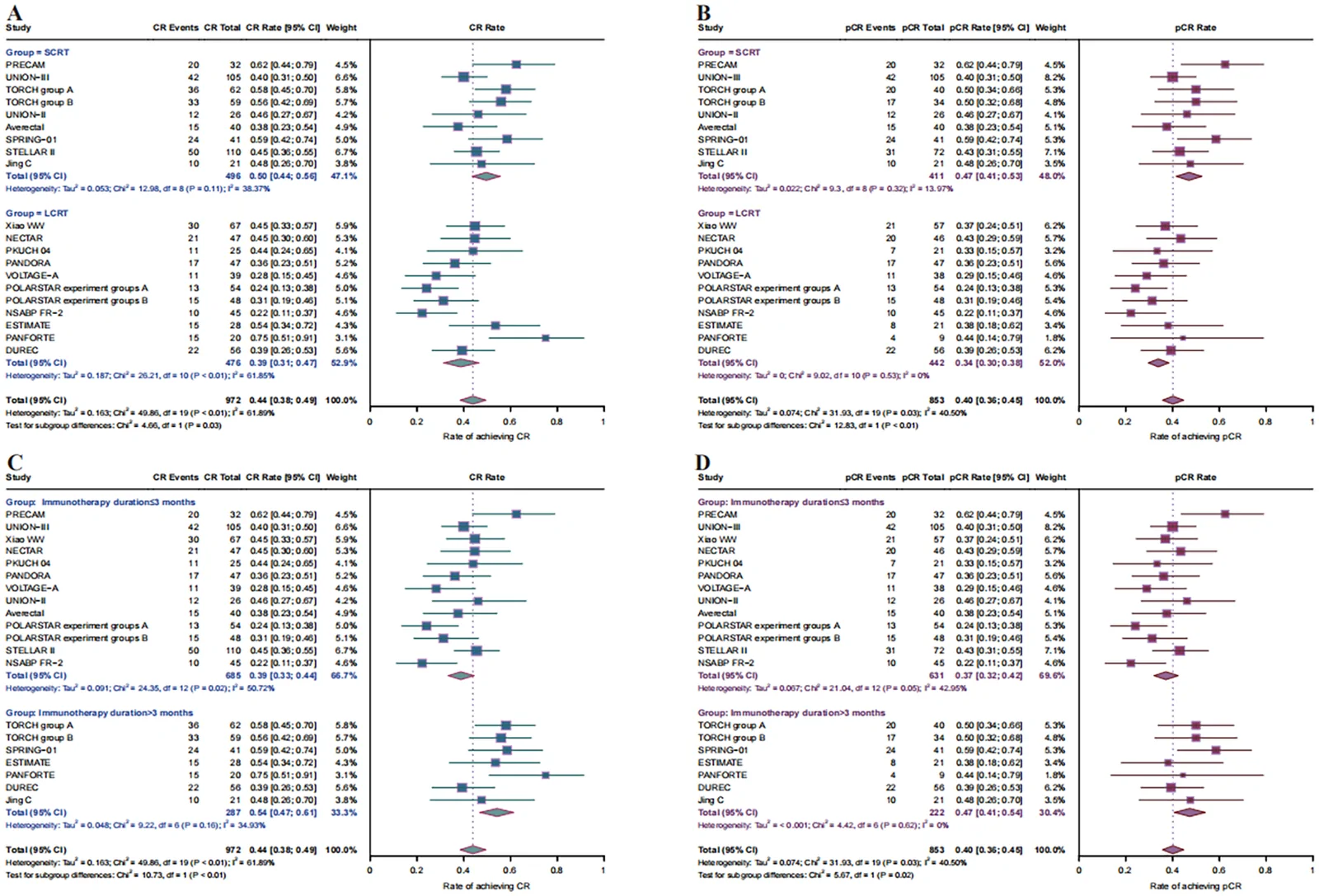
Forest plots of subgroup analysis based on different strategies. Forest plots illustrating subgroup analyses of CR and pCR rates in patients with pMMR LARC. **(A, B)** present subgroup analyses stratified by the type of radiotherapy combined with ICI. **(C, D)** depict subgroup analyses based on the duration of immunotherapy (≤3 months vs >3 months). For each study, squares represent the point estimate of the response rate, horizontal lines indicate the 95% *CI*, and the size of the square reflects the weight of the study in the meta-analysis. Diamonds represent pooled estimates with corresponding 95% CIs. Between-study heterogeneity was assessed using Tau², Cochran’s Q (Chi²) test, and the I² statistic. Subgroup differences were evaluated using Chi² tests, with corresponding *P* values reported in each panel. CR (cCR or pCR) indicates complete response (clinical complete response or pathological complete response); pCR, pathological complete response; SCRT, short-course radiotherapy; LCRT, long-course radiotherapy; ICI, immune checkpoint inhibitor; aPD-1, anti-programmed death-1; aPD-L1, anti–programmed death-ligand 1; pMMR/MSS, proficient mismatch repair or microsatellite-stable; LARC, locally advanced rectal cancer; *CI*, confidence interval.

We assessed the quality of the included full-text studies using the RoB-2 tool (42) for RCTs and the MINORS criteria (43) for non-randomized studies. Of the six included full-text RCTs, all were classified as having some concerns regarding the overall risk of bias (**Figure 2**). While all studies showed low risk of bias in domains D1 (randomization process) and D3 (missing outcome data), all six presented concerns in domain D2 (deviations from intended interventions). For the seven full-text non-randomized studies, MINORS scores ranged from 14 to 16 out of a maximum of 16 (**Figure 3**). All were considered high quality with a low risk of bias, as their total scores exceeded the 75% threshold established for the assessment. Quality assessment was not performed for the five included conference abstracts due to insufficient reporting of methodological details. Details of the quality assessment for full-text articles are provided in the **Supplementary File**.

Pooled estimates of efficacy outcomes across the included treatment arms are presented in **Figures 2A, B**. The pooled overall CR rate was 44% (95% *CI*, 0.38–0.49), with substantial between-study heterogeneity (*I*² = 61.89%). LOO sensitivity analysis demonstrated that the pooled CR estimate was stable, with recalculated CR rates ranging from 43% to 45% after sequential exclusion of individual studies, and no single study exerted a disproportionate influence on the overall result (**Supplementary Figure 4**). Heterogeneity estimates showed only modest fluctuations across sensitivity analyses, without qualitative changes in the overall degree of between-study variability.

**Figure 4:**
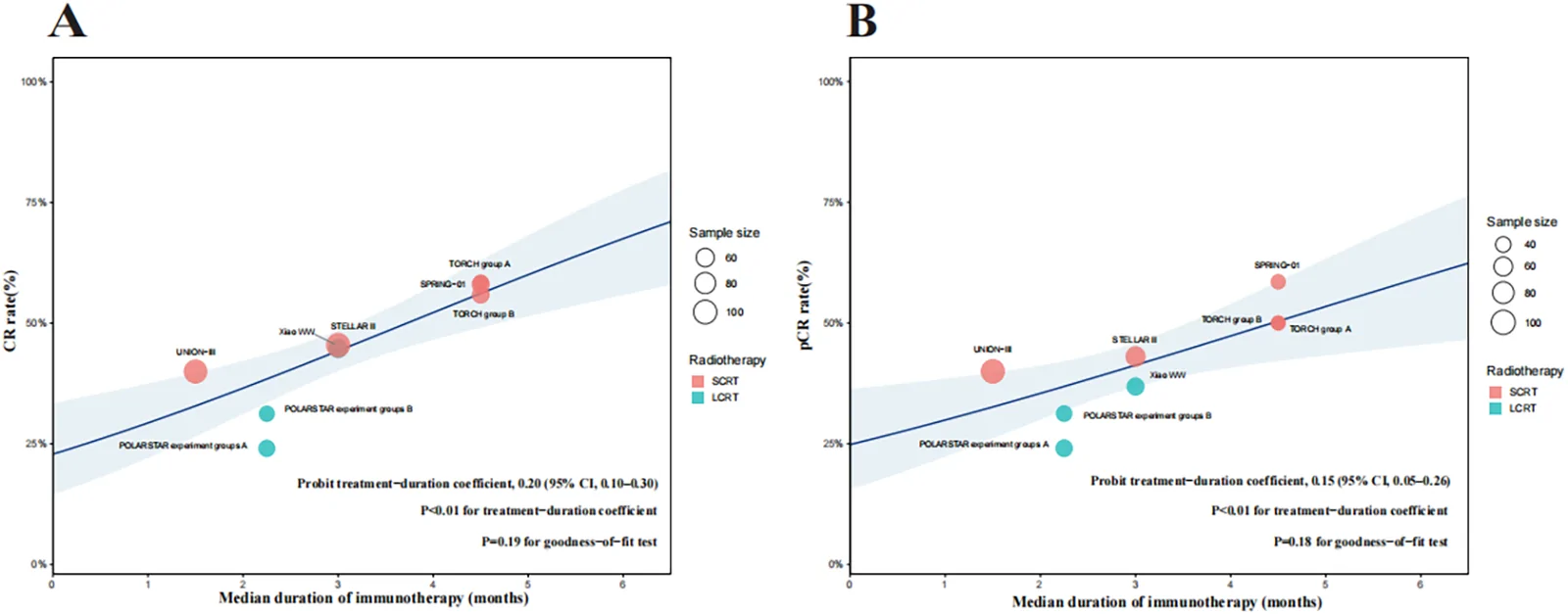
Linear association between duration of neoadjuvant PD-1/PD-L1 blockade and incidence of CR or pCR among pMMR/MSS LARC patients from RCTs. The figure shows the observed complete response rates in individual studies according to the median duration of neoadjuvant immunotherapy. **(A, B)** display the results from RCTs for CR and pCR rates, respectively. The red and teal circles represent SCRT and LCRT groups, respectively. The bubble size is weighted according to the sample size reported for each study. The solid blue lines represent the results of the linear probit regression model-fitting analysis, and the lighter shaded areas represent the 95% *CI*. A significant positive linear association was observed in the RCT group and the overall composite outcome cohort. The positive treatment-duration coefficients indicate that a longer duration of immunotherapy is associated with an increased probability of achieving a complete response. The specific coefficients were 0.20 (95% *CI*, 0.10–0.30; *P*<0.01) for CR , 0.15 (95% *CI*, 0.05–0.26; *P*<0.01) for pCR. The models for the RCT group were considered satisfactory as the *P* values for testing for goodness of fit were greater than 0.05. CR (cCR or pCR) indicates complete response (clinical complete response or pathological complete response); pCR, pathological complete response; SCRT, short-course radiotherapy; LCRT, long-course radiotherapy; pMMR/MSS, proficient mismatch repair or microsatellite-stable; LARC, locally advanced rectal cancer; PD-1, programmed death-1; PD-L1, programmed death-ligand 1; *CI*, confidence interval; RCTs, randomized controlled trials.

The pooled pCR rate was 40% (95% *CI*, 0.36–0.45). Compared with CR, heterogeneity for the pCR endpoint was lower (*I*² = 40.50%), indicating greater consistency across studies. Sensitivity analyses further confirmed the robustness of the pooled pCR estimate, with minimal variation observed across all exclusions, ranging from 39% to 41% (**Figure 4**). Heterogeneity for pCR consistently fell within the low-to-moderate range across all sensitivity analyses.

Subgroup analyses stratified by radiotherapy regimen revealed distinct efficacy patterns (**Figure 3A, B**). A test for subgroup differences showed a statistically significant difference in pooled overall CR rates between treatment strategies, with a higher pooled CR rate observed in the SCRT plus ICI group (50% [95% *CI*, 0.44–0.56]) compared with the LCRT plus ICI group (39% [95% *CI*, 0.31–0.47]; *P* = 0.03). Between-study heterogeneity was low in the SCRT subgroup (*I*² = 38.37%) and substantial in the LCRT subgroup (*I*² = 61.85%). Similarly, pooled pCR rates differed significantly between radiotherapy regimens based on the test for subgroup differences (*P* < 0.01). The SCRT subgroup demonstrated a higher pooled pCR rate (47% [95% *CI*, 0.41–0.54]) than the LCRT subgroup (34% [95% *CI*, 0.30–0.38]). Heterogeneity for the pCR endpoint was low in both subgroups (*I*² = 13.97% for SCRT and *I*² = 0% for LCRT), indicating high consistency of pCR estimates.

Subgroup analyses stratified by immunotherapy duration are shown in **Figures 3C, D**. Overall, higher pooled response rates were observed in studies in which immunotherapy was administered for more than 3 months compared with those with a treatment duration of 3 months or less. For CR, the pooled rate in the >3 months subgroup was 54% (95% *CI*, 0.47–0.61), compared with 39% (95% *CI*, 0.33–0.44) in the ≤3 months subgroup. Between-study heterogeneity was moderate in both subgroups (*I*² = 34.93% for >3 months and *I*² = 50.72% for ≤3 months). A similar pattern was observed for pCR. The pooled pCR rate was higher in the >3 months subgroup (47% [95% *CI*, 0.40–0.54]) than in the ≤3 months subgroup (37% [95% *CI*, 0.32–0.42]). Heterogeneity was negligible in the >3 months subgroup (*I*² = 0%) and low in the ≤3 months subgroup (*I*² = 42.95%).

In contrast, subgroup analyses stratified by ICIs showed no statistically significant differences in pooled CR or pCR rates between studies using anti–PD-1 and anti–PD-L1 inhibitors (**Supplementary Figure 5**).

Furthermore, univariate meta-regression was conducted to identify study-level factors associated with CR rate (**Table 2**). Regarding treatment parameters, the use of LCRT was inversely associated with CR rates compared to SCRT (coefficient, -0.46; 95% *CI*, -0.86–0.07; *P* = 0.02; *R²* = 36.9%). Longer immunotherapy duration was positively associated with CR (coefficient, 0.17; 95% *CI*, 0.01–0.34; *P* = 0.04; *R²* = 21.8%). No statistically significant associations were detected for the specific drug target (PD-1 vs. PD-L1) or the investigated baseline tumor burden parameters (proportions of cT4, cN+, cN2, EMVI+, and MRF/CRM+) (*P* > 0.05). These null findings should be interpreted cautiously because the analyses relied on aggregate data and may have had limited statistical power.

**Table 2:** Univariate meta-regression of treatment parameters and baseline tumor characteristics associated with pooled complete response.

Covariate	Coefficient (β_1_)	95% CI Lower	95% CI upper	P-value	R² (%)	Treatment arms (k)
Immune Duration	0.1727	0.0065	0.3389	0.042	21.84	20
RT Modality(LCRT)	-0.46441	-0.8598	-0.069	0.021	36.86	20
Drug Target(PD-L1)	-0.13414	-0.6253	0.357	0.592	0	20
cT4 (%)	0.00028	-0.0212	0.0218	0.979	0	16
cN+ (%)	0.0053	-0.009	0.0196	0.469	0	15
cN2 (%)	-0.00809	-0.0231	0.0069	0.29	0	15
EMVI+ (%)	0.00012	-0.013	0.0132	0.986	0	16
MRF/CRM+ (%)	0.00139	-0.0122	0.0149	0.841	0	15

CI, confidence interval; cN, clinical N; cT, clinical T; CRM, circumferential resection margin; EMVI, extramural vascular invasion; LCRT, long-course radiotherapy; MRF, mesorectal fascia; PD-L1, programmed death-ligand 1; RT, radiotherapy; k, number of treatment arms included in the meta-regression.

To evaluate the continuous association between immunotherapy duration and CR, we first restricted our analysis to a subset of six RCTs. Within this cohort, a parsimonious linear probit model yielded a satisfactory fit to the data (**Figure 4**). A significant positive linear association was observed between immunotherapy duration and CR rate (treatment–duration coefficient, 0.20; 95% *CI*, 0.10–0.30; *P* < 0.01), with acceptable model fit (goodness-of-fit, *P* = 0.19). Similar findings were observed for pCR, with a positive treatment–duration coefficient of 0.15 (95% *CI*, 0.05–0.26; *P* < 0.01) and satisfactory goodness-of-fit (*P* = 0.18).

**Figure 5:**
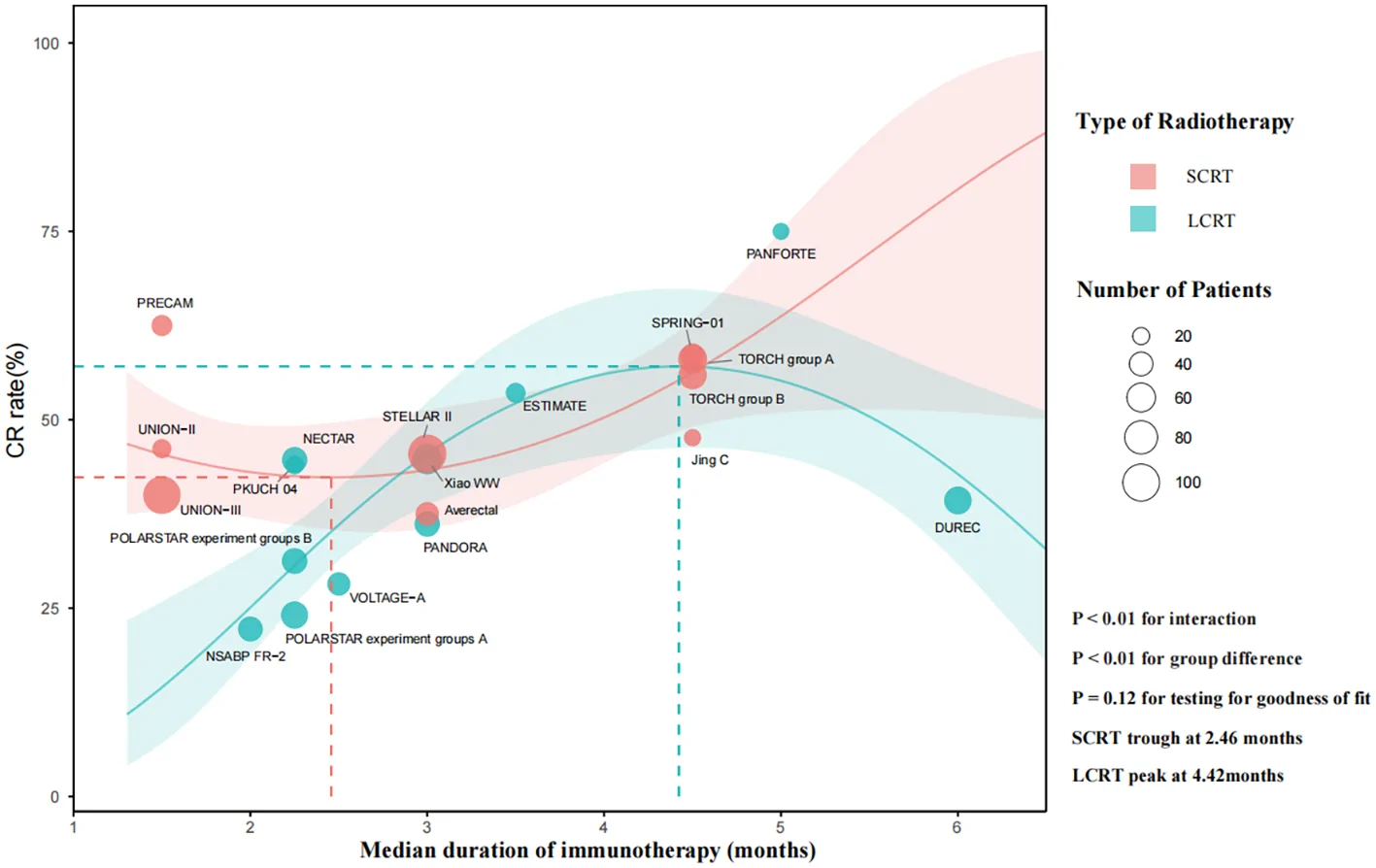
Nonlinear association between duration of neoadjuvant PD-1/PD-L1 blockade and incidence of CR among pMMR/MSS LARC patients. The figure shows the observed incidence of CR in individual studies according to the median duration of neoadjuvant PD-1/PD-L1 blockade in patients with pMMR/MSS LARC. The red and teal lines represent the results of the quadratic probit model-fitting analysis for SCRT and LCRT groups, respectively, and the lighter shaded areas represent the 95% confidence intervals. The LCRT group showed an inverted U-shaped relationship, with CR rates peaking at 4.42 months, whereas the SCRT group followed a U-shaped relationship, with a CR trough at 2.46 months. The analysis was weighted according to the sample size reported for each study. The *P* value for the interaction between immunotherapy duration and radiotherapy type, the *P* value for overall difference in complete response rate between SCRT and LCRT radiotherapy groups, and the *P* value for testing for goodness of fit are reported. The model was considered to be satisfactory as the *P* values for the interaction (*P* < 0.01) and overall comparison (*P* < 0.01) were less than 0.05, and the *P* value for testing for goodness of fit was more than 0.05 (*P* = 0.12). CR (cCR or pCR) indicates complete response (clinical complete response or pathological complete response); SCRT, short-course radiotherapy; LCRT, long-course radiotherapy; pMMR/MSS, proficient mismatch repair or microsatellite-stable; LARC, locally advanced rectal cancer; PD-1, programmed death-1; PD-L1, programmed death-ligand 1.

However, expanding the analysis to include all eligible studies resulted in an inadequate fit for linear modeling without stratification across the overall dataset (**Supplementary Figure 6**). Given the heterogeneity observed in the association between immunotherapy duration and CR, particularly across radiotherapy strategies, a flexible modeling framework was applied to further characterize the duration-response relationship. An interactive quadratic probit model incorporating immunotherapy duration, radiotherapy type, and their interaction, and weighted by study sample size, was fitted to the overall dataset to accommodate potential nonlinear effects (**Figure 5**).

The model demonstrated adequate fit to the observed data (goodness-of-fit *P* = 0.12) and identified a highly significant interaction between immunotherapy duration and radiotherapy strategy (*P* < 0.01), indicating that the duration–response relationship differed substantially between treatment regimens. Consistent with this finding, the likelihood ratio test supported stratification of the model by radiotherapy type (*P* < 0.01).

Within the stratified quadratic framework, distinct nonlinear efficacy patterns emerged. In the LCRT subgroup, the association followed an inverted U-shaped trajectory, with CR probability increasing with longer immunotherapy duration and reaching a maximum at approximately 4.42 months, followed by a decline with further prolongation of treatment. In contrast, the SCRT subgroup exhibited a U-shaped relationship, characterized by an initial decrease in CR probability with a nadir at approximately 2.46 months, followed by a subsequent recovery in response with extended immunotherapy exposure.

LOO sensitivity analyses were performed to evaluate the stability of the modeled relationships. First, the linear probit models, restricted exclusively to RCTs, demonstrated excellent stability for both CR and pCR (**Supplementary Figure 7**). Furthermore, when broadening the analysis to include all studies within an interactive quadratic probit model, the trajectories also showed acceptable consistency. In the SCRT subgroup, the U-shaped trajectory was strictly preserved across all exclusions, with only minor variations in curve magnitude and the location of the nadir (**Supplementary Figure 8**). In contrast, the LCRT subgroup results were sensitive to the DUREC study, the exclusion of which attenuated the inverted U-shaped curvature to a largely linear pattern. Nevertheless, the overall direction of the association remained consistent, and no single study exclusion led to a reversal of the observed trend (**Supplementary Figure 8**).

## 4 Discussion

Multiple prospective studies have suggested that integrating ICIs into neoadjuvant CRT may enhance complete response outcomes in patients with pMMR/MSS LARC. Notably, Wang et al (36) reported higher pCR rates with SCRT combined with ICIs compared with LCRT, implying that radiotherapy fractionation may meaningfully influence antitumor immune dynamics. Similarly, Ansab et al (35) observed improved tumor regression grades without excess toxicity following the addition of PD-1/PD-L1 inhibitors to CRT, further supporting the potential immunologic advantages of SCRT-based strategies. However, these prior analyses uniformly categorized immunotherapy duration into arbitrary intervals and did not evaluate duration as a continuous determinant of treatment efficacy. Although Rousseau et al (34) recently demonstrated a positive association between immunotherapy duration and CR in dMMR/MSI-H rectal cancer using a probit model, the relevance of such duration-response relationships to the more immune-resistant pMMR/MSS phenotype has remained uncertain.

This study evaluated the association between immunotherapy duration and clinical response in pMMR/MSS LARC. In analyses restricted to RCTs, longer immunotherapy duration was positively associated with CR and pCR. When all eligible prospective studies, including phase II studies with a single treatment arm, were incorporated, the linear model no longer provided an adequate fit. An exploratory quadratic model suggested an inverted U-shaped pattern for LCRT, with a modeled peak at approximately 4.42 months, and a U-shaped pattern for SCRT, with a modeled trough at approximately 2.46 months. These patterns may help describe heterogeneity across studies but may also reflect differences in study design, treatment regimens, and data density. They should therefore be interpreted as hypotheses rather than established radiobiological effects.

Additional support for this interpretation comes from the findings of Laengle et al. (45), who reported that augmenting LCRT with dual checkpoint blockade failed to meaningfully increase pCR or major pathologic response rates in pMMR/MSS rectal cancer, despite evidence of immunogenic remodeling within the tumor microenvironment. These observations are consistent with the possibility that the effect of checkpoint inhibition depends on treatment context rather than treatment intensity alone. However, they do not establish a specific window of immunotherapy susceptibility.

The mechanistic plausibility of the observed divergent duration-response patterns warrants further consideration. LCRT, delivered over a prolonged treatment course, is associated with sustained but relatively low-intensity antigen release while progressively inducing lymphocyte depletion (38, 46). Over time, dendritic-cell activation may diminish, antigen-presenting capacity may become impaired, and suppressive immune populations—such as regulatory T cells and myeloid-derived suppressor cells—may accumulate (47, 48). Concurrently, persistent antigen exposure and chronic inflammatory signaling can accelerate T-cell dysfunction and eventual exhaustion, aligning with the inverted U-shaped trajectory in which early immunotherapeutic benefit gives way to diminishing returns with extended immunotherapy (49, 50).

In contrast, SCRT induces a concentrated burst of immunogenic cell death accompanied by rapid antigen release and robust activation of innate immune pathways, including cGAS–STING signaling (39–41, 51). This acute immunogenic stimulus may create an optimal window for T-cell priming and subsequent expansion under PD-1/PD-L1 blockade. Although a transient inflammatory dip in response probability may occur shortly after SCRT, continued immunotherapy exposure may reinforce functional T-cell expansion and memory formation, providing a plausible explanation for the recovery phase of the U-shaped trajectory observed with longer treatment durations (52). Together, these radiobiological distinctions offer a unifying mechanistic framework through which immunotherapy duration and radiotherapy fractionation jointly shape treatment responsiveness in pMMR/MSS LARC.

From a clinical perspective, the modeled curves should not be used to determine immunotherapy duration. Instead, they generate the hypothesis that the association between immunotherapy duration and response may differ according to radiotherapy fractionation. Prospective trials with prespecified treatment durations and standardized background regimens are needed before these findings can inform clinical practice.

These exploratory interpretations must be weighed against the limitations of the available data. The distribution of immunotherapy duration within the LCRT subgroup was uneven (**Supplementary Figure 8**), with data points at the 5–6 month range derived primarily from the PANFORTE (24) and DUREC (17) cohorts. LOO sensitivity analyses confirmed that the LCRT model was sensitive to the inclusion of the DUREC study. Its exclusion attenuated the inverted U-shaped curvature to a largely linear pattern, although the overall positive direction of the association remained consistent. Consequently, the apparent peak in CR probability at approximately 4.42 months should be interpreted with caution, as it may partially reflect sparse data support at the upper end of the duration spectrum rather than a definitive biological cutoff. Prospective studies evaluating a wider range of treatment durations are needed to clarify the association in the LCRT setting.

This study has several methodological strengths. It evaluates immunotherapy duration as a continuous variable rather than only as a categorical exposure and explicitly examines its interaction with radiotherapy fractionation. It also synthesizes evidence from a broad range of prospective trials. These features provide an exploratory framework for generating hypotheses about variation in response across treatment regimens, while the heterogeneity of the included studies limits definitive interpretation.

Several limitations should be acknowledged. First, this analysis relied on aggregate data at the study level rather than individual patient data, limiting adjustment for important covariates such as baseline immune status, tumor mutational burden, and comorbidities. Immunotherapy duration was based on schedules specified in study protocols rather than actual treatment exposure among individual patients. Consequently, deviations from planned treatment due to adverse events or treatment adherence could not be captured. Clinical heterogeneity across studies, including differences in background treatment strategies (CRT versus TNT), chemotherapy backbones (such as FOLFOX and FOLFIRINOX), the timing of ICIs relative to CRT, and intervals to surgery or reassessment, may also have influenced tumor downstaging, immune activation, and response estimates. Given the limited number of studies and the use of aggregate data, we could not adequately adjust for these factors or conduct reliable subgroup analyses separating CRT from TNT within each duration model. The inclusion of randomized trials and phase II studies with a single treatment arm introduced further heterogeneity in study design, and efficacy estimates from the latter may be overestimated.

Second, survival outcomes were not quantitatively synthesized because the included studies were conducted in recent years and have not yet reported mature survival data. Quantitative synthesis was therefore restricted to CR and pCR, and the present findings should not be interpreted as evidence that longer ICI exposure improves survival. The modeled peaks and troughs represent exploratory statistical estimates rather than prescriptive clinical thresholds, and the proposed mechanistic explanations remain inferential. Longer follow-up and reporting of mature survival outcomes are needed to determine whether the observed associations translate into survival benefits.

## 5 Conclusion

Among the included studies, the association between immunotherapy duration and CR appeared to differ according to radiotherapy fractionation. Analyses restricted to RCTs showed a positive association, whereas exploratory models combining all prospective studies suggested different nonlinear patterns for LCRT and SCRT. Because these findings were based on aggregate data and may be affected by clinical and methodological heterogeneity, they should not be used to define treatment duration thresholds. Prospective trials are needed to validate these associations.

## Data Availability

The original contributions presented in the study are included in the article/**Supplementary Material**. Further inquiries can be directed to the corresponding author.
