# Supplementary material for: Nonlinear duration–efficacy association of neoadjuvant PD-1/PD-L1 blockade with chemoradiotherapy in pMMR/MSS locally advanced rectal cancer: a systematic review and meta-analysis
Source: Front Oncol. 2026 Sep 9;16:1855615. doi: 10.3389/fonc.2026.1855615 (PMC13597229; doi:10.3389/fonc.2026.1855615)
Supplement: Supplementary file 1 [file DataSheet1.docx]

**Figure S1. Assessment of Publication Bias for the CR and pCR Rate**

(A) Egger’s regression plot for the CR rate, showing the standardised treatment effect (z-score) against the inverse of the standard error. The solid line represents the linear regression fit. The results of Egger’s linear regression test (t=0.26, *P*=0.80) indicate no significant asymmetry. (B) Funnel plot for the CR rate, with the effect size (proportion) plotted against the standard error. Each circle represents an individual study included in the meta-analysis. The vertical dotted line represents the pooled estimate using the random-effects model, and the diagonal dotted lines represent the pseudo 95% confidence limits. (C) Egger’s regression plot for the pCR rate. The linear regression test results (t=0.08, *P*=0.93) demonstrate a lack of significant funnel plot asymmetry. (D) Funnel plot for the pCR rate. The symmetrical distribution of studies aligns with the non-significant Egger’s test result, suggesting no substantial publication bias.
Abbreviations: pCR, pathological complete response; cCR, clinical complete response; CR, complete response (including cCR and pCR).

**Figure S2. Risk of Bias Assessments for the Included Randomised Controlled Trials According to the ROB-2 Tool**

(A) Risk-of-bias summary illustrating review authors' judgments for each risk-of-bias item across all included studies. (B) Risk-of-bias graph illustrating review authors' judgments for each risk-of-bias item, presented as percentages across all included studies.

**Figure S3. Risk of Bias Assessment for the Included Single-Arm Trials and Non-Randomised Controlled Trial According to the MINORS Evaluation Criteria**

*For comparative studies only (Item 9-12).

Total scores of at least 75% were considered high quality with low risk for bias; scores between 50% and 75% were considered medium risk for bias; scores of less than or equal to 50% were considered high risk for bias. For noncomparative studies, the maximum score was 16, while the maximum score for comparative studies was 24.

**Figure S4. Leave-One-Out Sensitivity Analysis for CR and pCR Rates**

(A) Forest plot displaying the recalculated pooled CR rate after sequentially omitting each included study to assess individual study influence. The pooled estimate remained stable, indicating no single study had a disproportionate influence on the overall result. Heterogeneity (I²) fluctuated within a moderate range, with certain exclusions leading to notable reductions. (B) Forest plot showing the recalculated pooled pCR rate using the same leave-one-out approach. The pooled pCR rate demonstrated consistency across all exclusions. The exclusion of specific studies was associated with a meaningful decrease in statistical heterogeneity. CR (cCR or pCR) indicates complete response (clinical complete response or pathological complete response) ; pCR, pathological complete response; CI, confidence interval.

**Figure S5. Forest Plots of Subgroup Analysis Based on PD-1/PD-L1 Blockade.**

Forest plots illustrating subgroup analyses of CR and pCR rates in patients with pMMR LARC. For each study, squares represent the point estimate of the response rate, horizontal lines indicate the 95% *CI*, and the size of the square reflects the weight of the study in the meta-analysis. Diamonds represent pooled estimates with corresponding 95% CIs. Between-study heterogeneity was assessed using Tau², Cochran’s Q (Chi²) test, and the I² statistic. Subgroup differences were evaluated using Chi² tests, with corresponding *P* values reported in each panel. CR (cCR or pCR) indicates complete response (clinical complete response or pathological complete response); pCR, pathological complete response; aPD-1, anti-programmed death-1; aPD-L1, anti–programmed death-ligand 1; pMMR/MSS, proficient mismatch repair or microsatellite-stable; LARC, locally advanced rectal cancer; *CI*, confidence interval.

**Figure S6. Linear Association Between Duration of Neoadjuvant PD-1/PD-L1 Blockade and Incidence of CR Among pMMR/MSS LARC Patients.**

The figure shows the observed incidence of CR in individual studies according to the median duration of neoadjuvant PD-1/PD-L1 blockade in patients with pMMR/MSS LARC. Each point represents an individual study and is weighted according to the corresponding sample size. A linear probit regression model was applied to evaluate the association between immunotherapy duration and CR rate, with the fitted line representing the model-predicted CR probability and the lighter shaded area indicating the 95% confidence interval. A statistically significant positive association was observed between immunotherapy duration and CR rate, with a probit treatment-duration coefficient of 0.10 (95% CI, 0.04–0.17; *P* < 0.01). The *P* value for the goodness-of-fit test based on residual deviance is reported (*P* < 0.01), indicating potential model misspecification. Therefore, the fitted model should be interpreted as an exploratory description of the observed trend rather than a definitive predictive relationship. CR (cCR or pCR) indicates complete response (clinical complete response or pathological complete response); pMMR/MSS, proficient mismatch repair or microsatellite-stable; LARC, locally advanced rectal cancer; PD-1, programmed death-1; PD-L1, programmed death-ligand 1.

**Figure S7. Leave-One-Out Sensitivity Analysis of the Linear Probit Model**

The figure assesses the sensitivity of the model for the association between the median duration of neoadjuvant immunotherapy and the CR/pCR rate in patients with pMMR LARC. Each panel visually displays the impact on the model's predicted response rate curve when each listed study or defined set of studies is excluded. The lists of study names in each section (Dropped Set) represent the individual or grouped exclusions evaluated. CR (cCR or pCR) indicates complete response (clinical complete response or pathological complete response).

**Figure S8. Leave-One-Out Sensitivity Analysis of the Quadratic Probit Model**

The figure assesses the sensitivity of the model for the association between the median duration of neoadjuvant immunotherapy and the CR rate in patients with pMMR LARC, stratified by radiotherapy strategy. (A) and (B) present sensitivity analysis for SCRT group and LCRT group respectively. Each panel visually displays the impact on the model's predicted CR rate curve (on the probit scale) when each listed study or defined set of studies is excluded. The lists of study names in each section (Dropped Set) represent the individual or grouped exclusions evaluated. CR (cCR or pCR) indicates complete response (clinical complete response or pathological complete response); SCRT, short-course radiotherapy; LCRT, long-course radiotherapy.
