# Supplementary material for: Nonlinear duration–efficacy association of neoadjuvant PD-1/PD-L1 blockade with chemoradiotherapy in pMMR/MSS locally advanced rectal cancer: a systematic review and meta-analysis
Source: Front Oncol. 2026 Sep 9;16:1855615. doi: 10.3389/fonc.2026.1855615 (PMC13597229; doi:10.3389/fonc.2026.1855615)
Supplement: Supplementary file 2 [file Table1.docx]

**Supplementary Table S1: Prisma Checklist**

| **Section and Topic** | **Item #** | **Checklist item** | **Location where item is reported** |
| --- | --- | --- | --- |
| **TITLE** | | |  |
| Title | 1 | Identify the report as a systematic review. | Page1 |
| **ABSTRACT** | | |  |
| Abstract | 2 | See the PRISMA 2020 for Abstracts checklist. | Page2 |
| **INTRODUCTION** | | |  |
| Rationale | 3 | Describe the rationale for the review in the context of existing knowledge. | Page5 |
| Objectives | 4 | Provide an explicit statement of the objective(s) or question(s) the review addresses. | Page5-6 |
| **METHODS** | | |  |
| Eligibility criteria | 5 | Specify the inclusion and exclusion criteria for the review and how studies were grouped for the syntheses. | Page7 |
| Information sources | 6 | Specify all databases, registers, websites, organisations, reference lists and other sources searched or consulted to identify studies. Specify the date when each source was last searched or consulted. | Page6-7 |
| Search strategy | 7 | Present the full search strategies for all databases, registers and websites, including any filters and limits used. | Page6  Table S2 |
| Selection process | 8 | Specify the methods used to decide whether a study met the inclusion criteria of the review, including how many reviewers screened each record and each report retrieved, whether they worked independently, and if applicable, details of automation tools used in the process. | Page7-8 |
| Data collection process | 9 | Specify the methods used to collect data from reports, including how many reviewers collected data from each report, whether they worked independently, any processes for obtaining or confirming data from study investigators, and if applicable, details of automation tools used in the process. | Page7-8 |
| Data items | 10a | List and define all outcomes for which data were sought. Specify whether all results that were compatible with each outcome domain in each study were sought (e.g. for all measures, time points, analyses), and if not, the methods used to decide which results to collect. | Page7-8 |
|  | 10b | List and define all other variables for which data were sought (e.g. participant and intervention characteristics, funding sources). Describe any assumptions made about any missing or unclear information. | Page7-8 |
| Study risk of bias assessment | 11 | Specify the methods used to assess risk of bias in the included studies, including details of the tool(s) used, how many reviewers assessed each study and whether they worked independently, and if applicable, details of automation tools used in the process. | Page8 |
| Effect measures | 12 | Specify for each outcome the effect measure(s) (e.g. risk ratio, mean difference) used in the synthesis or presentation of results. | Page8-9 |
| Synthesis methods | 13a | Describe the processes used to decide which studies were eligible for each synthesis (e.g. tabulating the study intervention characteristics and comparing against the planned groups for each synthesis (item #5)). | Page7 |
|  | 13b | Describe any methods required to prepare the data for presentation or synthesis, such as handling of missing summary statistics, or data conversions. | Page7-8 |
|  | 13c | Describe any methods used to tabulate or visually display results of individual studies and syntheses. | Page8-9 |
|  | 13d | Describe any methods used to synthesize results and provide a rationale for the choice(s). If meta-analysis was performed, describe the model(s), method(s) to identify the presence and extent of statistical heterogeneity, and software package(s) used. | Page9 |
|  | 13e | Describe any methods used to explore possible causes of heterogeneity among study results (e.g. subgroup analysis, meta-regression). | Page9 |
|  | 13f | Describe any sensitivity analyses conducted to assess robustness of the synthesized results. | Page9 |
| Reporting bias assessment | 14 | Describe any methods used to assess risk of bias due to missing results in a synthesis (arising from reporting biases). | Page8  Figure S1 |
| Certainty assessment | 15 | Describe any methods used to assess certainty (or confidence) in the body of evidence for an outcome. | - |
| **RESULTS** | | |  |
| Study selection | 16a | Describe the results of the search and selection process, from the number of records identified in the search to the number of studies included in the review, ideally using a flow diagram. | Page10 |
|  | 16b | Cite studies that might appear to meet the inclusion criteria, but which were excluded, and explain why they were excluded. | - |
| Study characteristics | 17 | Cite each included study and present its characteristics. | Table 1 |
| Risk of bias in studies | 18 | Present assessments of risk of bias for each included study. | Page11  Figure S2-3 |
| Results of individual studies | 19 | For all outcomes, present, for each study: (a) summary statistics for each group (where appropriate) and (b) an effect estimate and its precision (e.g. confidence/credible interval), ideally using structured tables or plots. | Figure 2-5 |
| Results of syntheses | 20a | For each synthesis, briefly summarise the characteristics and risk of bias among contributing studies. | Page11-14 |
|  | 20b | Present results of all statistical syntheses conducted. If meta-analysis was done, present for each the summary estimate and its precision (e.g. confidence/credible interval) and measures of statistical heterogeneity. If comparing groups, describe the direction of the effect. | Page11-14 |
|  | 20c | Present results of all investigations of possible causes of heterogeneity among study results. | Page12-14 |
|  | 20d | Present results of all sensitivity analyses conducted to assess the robustness of the synthesized results. | Page12,16 |
| Reporting biases | 21 | Present assessments of risk of bias due to missing results (arising from reporting biases) for each synthesis assessed. | Figure S1 |
| Certainty of evidence | 22 | Present assessments of certainty (or confidence) in the body of evidence for each outcome assessed. | Figure 2-5 |
| **DISCUSSION** | | |  |
| Discussion | 23a | Provide a general interpretation of the results in the context of other evidence. | Page17-20 |
|  | 23b | Discuss any limitations of the evidence included in the review. | Page20-21 |
|  | 23c | Discuss any limitations of the review processes used. | Page22 |
|  | 23d | Discuss implications of the results for practice, policy, and future research. | Page20 |
| **OTHER INFORMATION** | | |  |
| Registration and protocol | 24a | Provide registration information for the review, including register name and registration number, or state that the review was not registered. | Page6 |
|  | 24b | Indicate where the review protocol can be accessed, or state that a protocol was not prepared. | PROSPERO |
|  | 24c | Describe and explain any amendments to information provided at registration or in the protocol. | - |
| Support | 25 | Describe sources of financial or non-financial support for the review, and the role of the funders or sponsors in the review. | Page10 |
| Competing interests | 26 | Declare any competing interests of review authors. | Page24 |
| Availability of data, code and other materials | 27 | Report which of the following are publicly available and where they can be found: template data collection forms; data extracted from included studies; data used for all analyses; analytic code; any other materials used in the review. | - |

**Supplementary Table S2: Detailed Search Strategy of Each Database**

| **Data Base** | **Search String** | **Results** |
| --- | --- | --- |
| PubMed | ("Rectal Neoplasms"[MeSH Terms] OR "neoplasm rectal"[Title/Abstract] OR "locally advanced rectal cancer"[Title/Abstract] OR "LARC"[Title/Abstract] OR "rectal neoplasm"[Title/Abstract] OR "neoplasms rectal"[Title/Abstract] OR "rectum neoplasms"[Title/Abstract] OR "neoplasm rectum"[Title/Abstract] OR "rectum neoplasm"[Title/Abstract] OR "rectal tumors"[Title/Abstract] OR "rectal tumor"[Title/Abstract] OR "tumor rectal"[Title/Abstract] OR "cancer of rectum"[Title/Abstract] OR "rectum cancers"[Title/Abstract] OR "cancer of the rectum"[Title/Abstract] OR "rectal cancer"[Title/Abstract] OR "cancer rectal"[Title/Abstract] OR "rectal cancers"[Title/Abstract] OR "rectum cancer"[Title/Abstract] OR "cancer rectum"[Title/Abstract])  AND  ("Immunotherapy"[MeSH Terms] OR "Immunotherapies"[Title/Abstract] OR "Immune Checkpoint Inhibitors"[MeSH Terms] OR "checkpoint inhibitors immune"[Title/Abstract] OR "immune checkpoint blockers"[Title/Abstract] OR "checkpoint blockers immune"[Title/Abstract] OR "immune checkpoint inhibitor"[Title/Abstract] OR "checkpoint inhibitor immune"[Title/Abstract] OR "ctla 4 inhibitors"[Title/Abstract] OR "ctla 4 inhibitors"[Title/Abstract] OR "cytotoxic t lymphocyte associated protein 4 inhibitors"[Title/Abstract] OR "cytotoxic t lymphocyte associated protein 4 inhibitors"[Title/Abstract] OR "cytotoxic t lymphocyte associated protein 4 inhibitor"[Title/Abstract] OR "cytotoxic t lymphocyte associated protein 4 inhibitor"[Title/Abstract] OR "ctla 4 inhibitor"[Title/Abstract] OR "ctla 4 inhibitor"[Title/Abstract] OR "pd 1 inhibitors"[Title/Abstract] OR "pd 1 inhibitors"[Title/Abstract] OR "programmed cell death protein 1 inhibitor"[Title/Abstract] OR "programmed cell death protein 1 inhibitors"[Title/Abstract] OR "pd 1 inhibitor"[Title/Abstract] OR "inhibitor pd 1"[Title/Abstract] OR "pd 1 inhibitor"[Title/Abstract] OR "immune checkpoint blockade"[Title/Abstract] OR "checkpoint blockade immune"[Title/Abstract] OR "immune checkpoint inhibition"[Title/Abstract] OR "checkpoint inhibition immune"[Title/Abstract] OR "pd l1 inhibitors"[Title/Abstract] OR "pd l1 inhibitors"[Title/Abstract] OR "programmed death ligand 1 inhibitors"[Title/Abstract] OR "programmed death ligand 1 inhibitors"[Title/Abstract] OR "pd l1 inhibitor"[Title/Abstract] OR "pd l1 inhibitor"[Title/Abstract] OR "pd 1 pd l1 blockade"[Title/Abstract] OR "blockade pd 1 pd l1"[Title/Abstract] OR "pd 1 pd l1 blockade"[Title/Abstract] OR "Camrelizumab"[Title/Abstract] OR "Tislelizumab"[Title/Abstract] OR "Toripalimab"[Title/Abstract] OR "Sintilimab"[Title/Abstract])  AND  ("Chemoradiotherapy"[MeSH Terms] OR ("Chemoradiotherapies"[Title/Abstract] OR "Radiochemotherapy"[Title/Abstract] OR "Radiochemotherapies"[Title/Abstract] OR "concurrent chemoradiotherapy"[Title/Abstract] OR (("Chemoradiotherapy"[MeSH Terms] OR "Chemoradiotherapy"[All Fields] OR "Chemoradiotherapies"[All Fields]) AND "Concurrent"[Title/Abstract]) OR "chemoradiotherapy concurrent"[Title/Abstract] OR "concurrent chemoradiotherapies"[Title/Abstract] OR "concomitant chemoradiotherapy"[Title/Abstract] OR (("Chemoradiotherapy"[MeSH Terms] OR "Chemoradiotherapy"[All Fields] OR "Chemoradiotherapies"[All Fields]) AND "Concomitant"[Title/Abstract]) OR "chemoradiotherapy concomitant"[Title/Abstract] OR (("concomitance"[All Fields] OR "Concomitant"[All Fields] OR "concomitants"[All Fields]) AND "Chemoradiotherapies"[Title/Abstract]) OR "concomitant radiochemotherapy"[Title/Abstract] OR (("concomitance"[All Fields] OR "Concomitant"[All Fields] OR "concomitants"[All Fields]) AND "Radiochemotherapies"[Title/Abstract]) OR (("Chemoradiotherapy"[MeSH Terms] OR "Chemoradiotherapy"[All Fields] OR "Radiochemotherapies"[All Fields]) AND "Concomitant"[Title/Abstract]) OR (("Chemoradiotherapy"[MeSH Terms] OR "Chemoradiotherapy"[All Fields] OR "Radiochemotherapy"[All Fields]) AND "Concomitant"[Title/Abstract]) OR "concurrent radiochemotherapy"[Title/Abstract] OR (("Concurrent"[All Fields] OR "concurrently"[All Fields] OR "concurrents"[All Fields]) AND "Radiochemotherapies"[Title/Abstract]) OR (("Chemoradiotherapy"[MeSH Terms] OR "Chemoradiotherapy"[All Fields] OR "Radiochemotherapies"[All Fields]) AND "Concurrent"[Title/Abstract]) OR (("Chemoradiotherapy"[MeSH Terms] OR "Chemoradiotherapy"[All Fields] OR "Radiochemotherapy"[All Fields]) AND "Concurrent"[Title/Abstract]) OR "synchronous chemoradiotherapy"[Title/Abstract] OR (("Chemoradiotherapy"[MeSH Terms] OR "Chemoradiotherapy"[All Fields] OR "Chemoradiotherapies"[All Fields]) AND "Synchronous"[Title/Abstract]) OR (("Chemoradiotherapy"[MeSH Terms] OR "Chemoradiotherapy"[All Fields] OR "Chemoradiotherapies"[All Fields]) AND "Synchronous"[Title/Abstract]) OR (("synchronic"[All Fields] OR "synchronically"[All Fields] OR "synchronicities"[All Fields] OR "synchronicity"[All Fields] OR "synchronisation"[All Fields] OR "synchronisations"[All Fields] OR "synchronise"[All Fields] OR "synchronised"[All Fields] OR "synchroniser"[All Fields] OR "synchronisers"[All Fields] OR "synchronises"[All Fields] OR "synchronising"[All Fields] OR "synchronism"[All Fields] OR "synchronisms"[All Fields] OR "synchronization"[All Fields] OR "synchronizations"[All Fields] OR "synchronize"[All Fields] OR "synchronized"[All Fields] OR "synchronizer"[All Fields] OR "synchronizers"[All Fields] OR "synchronizes"[All Fields] OR "synchronizing"[All Fields] OR "Synchronous"[All Fields] OR "synchronously"[All Fields]) AND "Chemoradiotherapies"[Title/Abstract])) OR ("Neoadjuvant Therapy"[MeSH Terms] OR ("neoadjuvant therapies"[Title/Abstract] OR "therapy neoadjuvant"[Title/Abstract] OR "neoadjuvant treatment"[Title/Abstract] OR "neoadjuvant treatments"[Title/Abstract] OR "treatment neoadjuvant"[Title/Abstract] OR "neoadjuvant chemotherapy"[Title/Abstract] OR "chemotherapy neoadjuvant"[Title/Abstract] OR "neoadjuvant chemotherapies"[Title/Abstract] OR "neoadjuvant chemotherapy treatment"[Title/Abstract] OR (("chemotherapy s"[All Fields] OR "drug therapy"[MeSH Terms] OR ("drug"[All Fields] AND "Therapy"[All Fields]) OR "drug therapy"[All Fields] OR "Chemotherapies"[All Fields] OR "drug therapy"[MeSH Subheading] OR "Chemotherapy"[All Fields]) AND "treatment neoadjuvant"[Title/Abstract]) OR "neoadjuvant chemotherapy treatments"[Title/Abstract] OR "treatment neoadjuvant chemotherapy"[Title/Abstract] OR "neoadjuvant chemoradiotherapy"[Title/Abstract] OR "chemoradiotherapy neoadjuvant"[Title/Abstract] OR "neoadjuvant chemoradiotherapies"[Title/Abstract] OR "neoadjuvant chemoradiation"[Title/Abstract] OR "chemoradiation neoadjuvant"[Title/Abstract] OR (("neoadjuvancy"[All Fields] OR "Neoadjuvant Therapy"[MeSH Terms] OR ("Neoadjuvant"[All Fields] AND "Therapy"[All Fields]) OR "Neoadjuvant Therapy"[All Fields] OR "Neoadjuvant"[All Fields] OR "neoadjuvants"[All Fields] OR "neoadjuvent"[All Fields]) AND "Chemoradiations"[Title/Abstract]) OR "neoadjuvant chemoradiation therapy"[Title/Abstract] OR "chemoradiation therapy neoadjuvant"[Title/Abstract] OR "neoadjuvant chemoradiation therapies"[Title/Abstract] OR "therapy neoadjuvant chemoradiation"[Title/Abstract] OR "neoadjuvant chemoradiation treatment"[Title/Abstract] OR (("Chemoradiotherapy"[MeSH Terms] OR "Chemoradiotherapy"[All Fields] OR "Chemoradiation"[All Fields]) AND "treatment neoadjuvant"[Title/Abstract]) OR (("neoadjuvancy"[All Fields] OR "Neoadjuvant Therapy"[MeSH Terms] OR ("Neoadjuvant"[All Fields] AND "Therapy"[All Fields]) OR "Neoadjuvant Therapy"[All Fields] OR "Neoadjuvant"[All Fields] OR "neoadjuvants"[All Fields] OR "neoadjuvent"[All Fields]) AND "chemoradiation treatments"[Title/Abstract]) OR "treatment neoadjuvant chemoradiation"[Title/Abstract] OR "neoadjuvant systemic therapy"[Title/Abstract] OR "neoadjuvant systemic therapies"[Title/Abstract] OR "systemic therapy neoadjuvant"[Title/Abstract] OR "therapy neoadjuvant systemic"[Title/Abstract] OR "neoadjuvant systemic treatment"[Title/Abstract] OR "neoadjuvant systemic treatments"[Title/Abstract] OR "systemic treatment neoadjuvant"[Title/Abstract] OR (("therapeutics"[MeSH Terms] OR "therapeutics"[All Fields] OR "Treatments"[All Fields] OR "Therapy"[MeSH Subheading] OR "Therapy"[All Fields] OR "Treatment"[All Fields] OR "treatment s"[All Fields]) AND "neoadjuvant systemic"[Title/Abstract]) OR "neoadjuvant radiotherapy"[Title/Abstract] OR (("neoadjuvancy"[All Fields] OR "Neoadjuvant Therapy"[MeSH Terms] OR ("Neoadjuvant"[All Fields] AND "Therapy"[All Fields]) OR "Neoadjuvant Therapy"[All Fields] OR "Neoadjuvant"[All Fields] OR "neoadjuvants"[All Fields] OR "neoadjuvent"[All Fields]) AND "Radiotherapies"[Title/Abstract]) OR "radiotherapy neoadjuvant"[Title/Abstract] OR "neoadjuvant radiation"[Title/Abstract] OR (("neoadjuvancy"[All Fields] OR "Neoadjuvant Therapy"[MeSH Terms] OR ("Neoadjuvant"[All Fields] AND "Therapy"[All Fields]) OR "Neoadjuvant Therapy"[All Fields] OR "Neoadjuvant"[All Fields] OR "neoadjuvants"[All Fields] OR "neoadjuvent"[All Fields]) AND "Radiations"[Title/Abstract]) OR "radiation neoadjuvant"[Title/Abstract] OR "neoadjuvant radiation therapy"[Title/Abstract] OR (("neoadjuvancy"[All Fields] OR "Neoadjuvant Therapy"[MeSH Terms] OR ("Neoadjuvant"[All Fields] AND "Therapy"[All Fields]) OR "Neoadjuvant Therapy"[All Fields] OR "Neoadjuvant"[All Fields] OR "neoadjuvants"[All Fields] OR "neoadjuvent"[All Fields]) AND "radiation therapies"[Title/Abstract]) OR "radiation therapy neoadjuvant"[Title/Abstract] OR (("therapeutics"[MeSH Terms] OR "therapeutics"[All Fields] OR "Therapies"[All Fields] OR "Therapy"[MeSH Subheading] OR "Therapy"[All Fields] OR "therapy s"[All Fields] OR "therapys"[All Fields]) AND "neoadjuvant radiation"[Title/Abstract]) OR "neoadjuvant radiation treatment"[Title/Abstract] OR (("neoadjuvancy"[All Fields] OR "Neoadjuvant Therapy"[MeSH Terms] OR ("Neoadjuvant"[All Fields] AND "Therapy"[All Fields]) OR "Neoadjuvant Therapy"[All Fields] OR "Neoadjuvant"[All Fields] OR "neoadjuvants"[All Fields] OR "neoadjuvent"[All Fields]) AND "radiation treatments"[Title/Abstract]) OR "radiation treatment neoadjuvant"[Title/Abstract] OR (("therapeutics"[MeSH Terms] OR "therapeutics"[All Fields] OR "Treatments"[All Fields] OR "Therapy"[MeSH Subheading] OR "Therapy"[All Fields] OR "Treatment"[All Fields] OR "treatment s"[All Fields]) AND "neoadjuvant radiation"[Title/Abstract])))) | 186 results (08/10/2025) |
| Cochrane Central Library | ("Rectal Neoplasms" OR "rectal neoplasm" OR "rectal neoplasms" OR "rectum neoplasm" OR "rectum neoplasms" OR "rectal tumor" OR "rectal tumors" OR "rectal cancer" OR "rectal cancers" OR "rectum cancer" OR "rectum cancers" OR "cancer of rectum" OR "cancer of the rectum" OR "locally advanced rectal cancer" OR LARC) in Title Abstract Keyword  AND  ("Immunotherapy" OR Immunotherapy OR Immunotherapies OR "Immune Checkpoint Inhibitors" OR "Immune Checkpoint Inhibitor" OR "Immune Checkpoint Blockers" OR "Immune Checkpoint Blockade" OR "Immune Checkpoint Inhibition" OR "CTLA-4 Inhibitors" OR "CTLA-4 Inhibitor" OR "CTLA 4 Inhibitors" OR "CTLA 4 Inhibitor" OR "Cytotoxic T-Lymphocyte-Associated Protein 4 Inhibitors" OR "Cytotoxic T-Lymphocyte-Associated Protein 4 Inhibitor" OR "Cytotoxic T Lymphocyte Associated Protein 4 Inhibitors" OR "Cytotoxic T Lymphocyte Associated Protein 4 Inhibitor" OR "PD-1 Inhibitors" OR "PD-1 Inhibitor" OR "PD 1 Inhibitors" OR "PD 1 Inhibitor" OR "Programmed Cell Death Protein 1 Inhibitor" OR "Programmed Cell Death Protein 1 Inhibitors" OR "PD-L1 Inhibitors" OR "PD-L1 Inhibitor" OR "PD L1 Inhibitors" OR "PD L1 Inhibitor" OR "Programmed Death-Ligand 1 Inhibitors" OR "Programmed Death Ligand 1 Inhibitors" OR "PD-1-PD-L1 Blockade" OR "PD 1 PD L1 Blockade" OR Camrelizumab OR Tislelizumab OR Toripalimab OR Sintilimab) in Title Abstract Keyword  AND  ("Neoadjuvant Therapy" OR "Neoadjuvant Therapy" OR "Neoadjuvant Therapies" OR "Neoadjuvant Treatment" OR "Neoadjuvant Treatments" OR "Neoadjuvant Chemotherapy" OR "Neoadjuvant Chemotherapies" OR "Neoadjuvant Chemotherapy Treatment" OR "Neoadjuvant Chemotherapy Treatments" OR "Neoadjuvant Chemoradiotherapy" OR "Neoadjuvant Chemoradiotherapies" OR "Neoadjuvant Chemoradiation" OR "Neoadjuvant Chemoradiations" OR "Neoadjuvant Chemoradiation Therapy" OR "Neoadjuvant Chemoradiation Therapies" OR "Neoadjuvant Chemoradiation Treatment" OR "Neoadjuvant Chemoradiation Treatments" OR "Neoadjuvant Systemic Therapy" OR "Neoadjuvant Systemic Therapies" OR "Neoadjuvant Systemic Treatment" OR "Neoadjuvant Systemic Treatments" OR "Neoadjuvant Radiotherapy" OR "Neoadjuvant Radiotherapies" OR "Neoadjuvant Radiation" OR "Neoadjuvant Radiations" OR "Neoadjuvant Radiation Therapy" OR "Neoadjuvant Radiation Therapies" OR "Neoadjuvant Radiation Treatment" OR "Neoadjuvant Radiation Treatments" OR "Chemoradiotherapy" OR "Chemoradiotherapies" OR "Radiochemotherapy" OR "Radiochemotherapies" OR "Concurrent Chemoradiotherapy" OR "Concurrent Chemoradiotherapies" OR "Concomitant Chemoradiotherapy" OR "Concomitant Chemoradiotherapies" OR "Concomitant Radiochemotherapy" OR "Concomitant Radiochemotherapies" OR "Concurrent Radiochemotherapy" OR "Concurrent Radiochemotherapies" OR "Synchronous Chemoradiotherapy" OR "Synchronous Chemoradiotherapies") | 105 results (08/10/2025) |
| Embase | ('rectum tumor'/exp OR ((('mass, rectum':ab,ti AND tumor:ab,ti OR 'neoplasm of the rectum':ab,ti OR 'neoplasma recti':ab,ti OR 'pararectal tumor':ab,ti OR 'pararectal tumour':ab,ti OR 'rectal mass':ab,ti) AND tumor:ab,ti OR 'rectal neoplasia':ab,ti OR 'rectal neoplasm':ab,ti OR 'rectal neoplasms':ab,ti OR 'rectal tumor':ab,ti OR 'rectal tumour':ab,ti OR 'rectum mass':ab,ti) AND tumor:ab,ti) OR 'rectum neoplasia':ab,ti OR 'rectum neoplasm':ab,ti OR 'rectum tumour':ab,ti OR 'retrorectal tumor':ab,ti OR 'retrorectal tumour':ab,ti OR 'tumor of the rectum':ab,ti OR 'tumor recti':ab,ti OR 'tumour of the rectum':ab,ti OR 'tumour recti':ab,ti OR 'rectum tumor':ab,ti OR 'locally advanced rectal cancer':ab,ti OR 'larc':ab,ti)  AND  ('chemoradiotherapy'/exp OR 'chemoradiation':ab,ti OR 'radiochemotherapy':ab,ti OR 'chemoradiotherapy':ab,ti OR 'neoadjuvant therapy'/exp OR 'neo-adjuvant drug therapy':ab,ti OR 'neo-adjuvant drug treatment':ab,ti OR 'neo-adjuvant medication':ab,ti OR 'neo-adjuvant therapy':ab,ti OR 'neo-adjuvant treatment':ab,ti OR 'neoadjuvant drug therapy':ab,ti OR 'neoadjuvant drug treatment':ab,ti OR 'neoadjuvant medication':ab,ti OR 'neoadjuvant treatment':ab,ti OR 'neoadjuvant therapy':ab,ti)  AND  ('immune checkpoint inhibitor'/exp OR 'immune checkpoint blocker':ab,ti OR 'immune checkpoint inhibitors':ab,ti OR 'immune checkpoint inhibitor':ab,ti OR 'immunotherapy'/exp OR 'biologic response modifier therapy':ab,ti OR 'biological response modifier therapy':ab,ti OR 'brm therapy':ab,ti OR 'immune therapy':ab,ti OR 'immunogenic therapy':ab,ti OR 'immunoglobulin therapy':ab,ti OR 'immunological therapy':ab,ti OR 'immunological treatment':ab,ti OR 'immunomodulant therapy':ab,ti OR 'immunomodulary therapy':ab,ti OR 'immunomodulating therapy':ab,ti OR 'immunomodulation therapy':ab,ti OR 'immunomodulative therapy':ab,ti OR 'immunomodulator therapy':ab,ti OR 'immunomodulatory intervention':ab,ti OR 'immunomodulatory therapy':ab,ti OR 'immunomoduling therapy':ab,ti OR 'immunomodurating therapy':ab,ti OR 'immunotherapy':ab,ti) | 730  results  08/10/2025 |
